# Sex-specific behavioral flexibility in rapid adaptation to a new environment

**DOI:** 10.1186/s12983-025-00586-y

**Published:** 2025-11-04

**Authors:** Marko Glogoški, Tomislav Gojak, Duje Lisičić, Ivan Cizelj, Iva Sabolić, Anamaria Štambuk

**Affiliations:** 1 Department of Biology, Faculty of Science, University of Zagreb, Rooseveltov trg 6, 10000 Zagreb, Croatia; 2 Association Hyla, Lipovac I 7, 10000 Zagreb, Croatia; 3 Zoological Garden of Zagreb, Ul. Fakultetsko dobro 1, Zagreb, Croatia

**Keywords:** Lizards, Behavior, *Podarcis siculus*, Behavioral sexual dimorphism, Activity, Exploratory behavior, Boldness, Founder effect

## Abstract

**Background:**

Behavioral adaptations are considered an important factor of population success in colonizing novel environments. Individuals can be selected for specific behavioral traits during transport, introduction and especially establishment phase of the invasion process. Aside from population level average of behavioral traits, both among- and within individual variability can contribute to achieving behavioral optima for efficiently acquiring resources in new habitats. Here, we study activity/exploration behavioral traits and boldness in a novel insular population of Italian wall lizard (*Podarcis siculus*) with a known colonizing history and propagule pressure. We apply Bayesian mixed-effects models and variance partitioning to compare the activity/exploration behavioral traits and boldness between ancestral population from Pod Kopište island and novel population from Pod Mrčaru island.

**Results:**

We found no difference in average levels of activity/exploration behavioral traits (distance moved and angular velocity) or boldness between populations or sexes. Among-individual variance in both behaviors was preserved in novel population, despite small propagule size of ten individuals. Females from ancestral Pod Kopište had substantially lower within-individual variability of distance moved than males. However, females within-individual variability for this trait increased in the novel Pod Mrčaru population, while males remained the same. Females on Pod Mrčaru population also exhibited strong increase in within-individual variability in angular velocity, even surpassing the values denoted for males in that population. In contrast, within-individual variance in boldness did not differ across population by sex groups.

**Conclusions:**

Our results show that among-individual behavioral variation can be preserved even in populations founded by small propagule. Our study also demonstrates sexual dimorphism in the within-individual variability of activity/exploration behavioral traits, both within the populations and in the direction and intensity of change in a new environment. Collectively, this study highlights the importance of studying behavioral flexibility in addition to average population or individual behavioral traits and emphasizes the role of females’ activity/exploration in adaptation to new environments.

**Supplementary Information:**

The online version contains supplementary material available at 10.1186/s12983-025-00586-y.

## Background

Studying the behavior of species with great invasive potential can help researchers predict their potential success and impact on new ecosystems [1]. During the colonization of new environments naturally or via human-mediated transport, the selective filtering of behavioral traits can occur at different stages, i.e., transport, introduction or establishment [1]. For example, more exploratory and bolder animals can have a greater chance of being transported into a novel environment [2, 3]. Hence, during this stage, individuals can be differentially selected according to their propensity to be transported [4], which can directly influence the behavioral traits available within the founding population. During their introduction into new environments, animals often need to obtain resources (e.g., food, shelter and mates), whose quantity, quality and distribution may differ from those in their original environments. Behavioral adaptations can help populations adjust to these challenges. For example, greater exploratory behavior can modify or broaden the population ecological niche through the utilization of novel food sources, shelters, or habitats, facilitating successful introduction and establishment in new environments [5–10]. However, in the presence of increased predation, individuals who are more exploratory and/or bold can be more exposed to predators, which can inflict detrimental and even lethal outcomes [11, 12]. During the establishment phase, the ecological conditions in the novel environment can further independently act as selective factors toward specific behavioral traits [13].

Behavioral variance, encompassing both the variability among and within individuals in a population, can additionally promote rapid adaptation to altered environmental conditions or new environments [4, 14–16]. Notably, the source of trait variation within populations can have distinctive ecological and biological significance [17]. Even when two populations have the same mean value of a behavioral trait, populations can differ in terms of the amount of among- and within-individual behavioral variance. For example, one population may exhibit a high degree of variability among individuals, who are consistent in their behavioral performance. At the same time, the other population might have lower variability among individuals, with each individual having high within-individual variability [17]. Invasive populations often respond to novel conditions with an increase in within-individual variation in behaviors [10, 13, 18]. Behavioral flexibility can be broadly defined as change in behavior driven by external or internal environment, and is often measured as within-individual behavioral variance [19–21]. In research on behavioral flexibility, it is important to examine different behavioral traits because particular traits can impact success in the colonization of new environments in distinct ways [4]. The most frequently investigated behaviors are exploratory behavior, activity, and boldness because they can directly influence multiple stages of invasion, i.e., transport, introduction, and establishment [4, 13]. Because activity and exploration are often highly intercorrelated and sometimes difficult to distinguish empirically in experimental assays, we refer to those behaviors collectively as 'activity/exploration' [22–24].

Successful colonization of new environments is also influenced by propagule pressure, i.e., the frequency and magnitude of the introduction of new individuals to the environment [25, 26]. Propagules may be composed of a single individual [27, 28] or small groups [25, 29], which may vary with respect to source habitat, introduction route and timing [4]. Given the importance of propagule quality and quantity for determining phenotypic variation in newly established populations, it is highly advantageous to study behavioral traits in systems with well-known colonization histories.

In 1971, Nevo and colleagues created an experimental model by conducting a reciprocal transplant experiment in the Lastovo Archipelago (Adriatic Sea) between two small islands: Pod Mrčaru, which was inhabited by the Dalmatian wall lizard (*Podarcis melisellensis* (Braun, 1877)), and Pod Kopište, which was inhabited by the Italian wall lizard (*Podarcis siculus* (Rafinesque-Schmaltz, 1810)) [30] (Fig. S1). The experiment included transferring randomly chosen five adult pairs of *P. siculus* from Pod Kopište to Pod Mrčaru and five pairs of *P. melisellensis* from Pod Mrčaru to Pod Kopište. Both lizard species are native to the area and have similar morphological appearances and ecological requirements. However, there is one major difference: *P. melisellensis* is endemic to Eastern Adriatic, whereas *P. siculus* is a highly adaptable species that thrives in urban environments and shows invasive potential, with introduced populations in Greece, Turkey, Portugal, the United Kingdom, and the USA [31, 32].

After 36 years, *P. siculus* was the only lizard species found on both islands, confirming the competitive exclusion of *P. melisellensis* [33, 34]*.* The new population of *P. siculus* on Pod Mrčaru showed differences in morphology, performance, and behavior [33, 34]. Both islands have similar climatic and geological conditions but vary in terms of vegetation, prey diversity and predator pressure [34]. In Pod Mrčaru, there is only one plant community, whereas Pod Kopište vegetation is characterized by two plant communities and overall higher floristic and prey diversity [35]. In addition, on Pod Mrčaru, the population abundance of *P. siculus* is greater, whereas the predator pressure is somewhat lower than that on Pod Kopište [33, 34].

This research system is characterized by 1) a highly adaptable species with a known history of being invasive; 2) a documented propagule event that occurred in 1971 [30]; 3) similar climatological conditions and different biotic factors between environments; and 4) a founder event that has not resulted in a strong bottleneck effect [35]. Our previous study further confirmed that no further introduction has occurred since the initial settlement [35]. This exceptional system thus provides a rare opportunity to study how a novel environment shapes behavior and behavioral plasticity within the context of known propagule pressure and a lack of selection during the transport stage.

Here, we compare the behaviors of the newly established population from Pod Mrčaru island with those of an ancestral population of *P. siculus* from Pod Kopište island. We measured activity/exploration behavioral traits and boldness across three different contexts to assess behavioral plasticity for each population. Specifically, our aims were to determine the following:

1. Does the activity/exploration behavioral traits and boldness of a newly established *P. siculus* population on Pod Mrčaru differ from that of the original population on Pod Kopište?
  We hypothesized that the population in a new environment would exhibit higher levels of activity/exploration behavior, which might facilitate the utilization of novel resources and widen the dietary niche. We hypothesized that boldness would increase on Pod Mrčaru island in accordance with the lower predation pressure.
2. Are there differences in behavioral variability among and within individuals between the populations of *P. siculus* in the new and ancestral habitats?
  We hypothesized that the population in a new environment will exhibit lower variation among individuals because of a founder event. We further predict that within-individual behavioral variability (flexibility) in activity/exploration behavioral traits will increase in response to novel ecological conditions, whereas there will be no change in within-individual variability in boldness.

## Methods

### Sampling and maintenance of animals

Lizards were sampled in March 2017 and 2018 on both islands. The area of Pod Kopište is 35,835 m^2^ and the area of Pod Mrčaru 13,514 m^2^. We tested the behavior of 27 females and 21 males from Pod Kopište and 16 females and 24 males from Pod Mrčaru (88 lizards in total). The animals were brought to the Zoological Garden of Zagreb, and following the 4-week acclimatization period, one male and one female from the same island were paired and placed in terrariums until September of the year of capture (for the purpose of another experiment, common garden crossing). The lizards were housed in glass or plastic terrariums (60 × 30 × 30 cm) with a peat substrate, a UV lamp, a heating bulb, basking rocks, dried bark, and plastic containers filled with vermiculite for hiding and laying eggs. The light cycle was set to 12:12 (light:dark), with daytime temperatures ranging from 23–24 °C and nighttime temperatures of 20 °C. Lizards were allowed to freely thermoregulate underneath the heating bulb. The lizards were fed crickets (*Gryllus assimilis*) supplemented with calcium and vitamins. In September, the lizards were transferred to the testing facility at the Department of Biology, Faculty of Science, University of Zagreb, and allowed to acclimatize for two weeks. Here, the lizards were held in individual plastic terrariums (40 × 30 cm), with the inner terrarium environment and room conditions being the same as those in the Zoo facility.

### Behavioral tests

After the acclimatization period, all lizards (27 females and 21 males from Pod Kopište and 16 females and 24 males from Pod Mrčaru) underwent behavioral tests in three different contexts (novel environment, familiar environment, familiar environment with novel object) over two consecutive days. All tests were conducted in a room specially designed for behavioral testing. Lizard behavior was tested in the open field arena, which was an empty square opaque Plexiglas box (50 × 50 cm and 50 cm high) with an open top including a central and peripheral zone (defined as 10 cm from each wall of the open field; Fig. S2).

Activity/exploration behavioral traits and boldness were measured in three contexts: 1) in the novel environment, 2) in the familiar environment, and 3) in the familiar environment with a novel object. On the first day, each lizard was introduced twice into an open field arena. During the first exposure, activity/exploration behavior and boldness in a novel environment were measured. The second exposure to an open field served only to familiarize lizards with the apparatus under the same conditions as in the first trial, and no behaviors were measured. On the second day, two tests were performed in the open field: first, activity/exploration behavior and boldness in the familiar environment were measured in the same way as they were on the first day. During the second test, a novel object (a small piece of rubber glove without powder tied into a knot) was placed in the center of the arena, and activity/exploration behavioral traits and boldness were measured.

The temperature in the experimental room was maintained at 30 °C to stimulate lizard activity [36, 37]. Before each experiment, the test chamber was cleaned with 30% alcohol to eliminate any potential odors [38, 39]. All tests lasted 15 min and were recorded with a camera from above the apparatus. The videos were analyzed via the Noldus EthoVision XT 15 program (Noldus, Wageningen, Netherlands).

Distance moved and angular velocity were scored as measures of activity/exploration behavioral traits, whereas time spent in a central zone was scored as a measure of boldness across all three contexts: a novel environment (day one), a familiar environment (day two) and a familiar environment with a novel object (day two) (Table 1). These three contexts were needed to assess the repeatability and within- and among-individual variation. The direct interactions of lizards with novel objects were not analyzed due to a lack of repeatable measurements of this behavior. The order in which the lizards entered the experiment was randomized with an online program (https://www.randomizer.org). The lizards in the second test entered the experiment in the same order as those in the first test did, resulting in the same time interval between the two tests for each lizard.

**Table 1:** Variables scored for each type of behavior

Type of behavior	Variables scored from videos (measure unit)	Definition
Activity/exploration	Distance moved (cm)	Total distance the animal moved during a trial
	Angular velocity (rad/s)	The rate of change in direction of the lizard's movement during a trial
Boldness	Central zone (s)	Total time spent in the central zone (open space)

### Statistical analysis

We employed Bayesian mixed-effects models, with separate models fitted for each behavioral variable (distance moved, angular velocity, and central zone) to estimate the average level of trait expression and trait variation [17, 40, 41]. The first part of the model estimated average behavioral levels included fixed effects for population (Pod Kopište/Pod Mrčaru), sex (male/female), their interaction, and trial number (context of tests). Individual ID was included as a random effect to account for repeated measures. In the second part, we compared the fits of four competing models:

- **Model 1 (Null Model)**: Assumes constant among-individual variance (VA) and within-individual variance (VW) across populations by sex groups
- **Model 2 (Among-individual Variance Model)**: Assumes that among-individual variance (VA) varies by location, but within-individual variance remains constant across populations by sex groups
- **Model 3 (Within-individual Variance Model)**: Assumes that within-individual variance (VW) varies by population by sex groups, while among-individual variance remains constant
- **Model 4 (Variance Model)**: Allows both among-individual variance (VA) and within-individual variance (VW) to vary across population by sex groups

Random effects were modeled as random intercepts per individual for models 1 and 3 and as group-specific slopes for models 2 and 4 to capture how individuals might differ in their response patterns. In models 1 and 2, the residual variance was assumed to be constant across all groups. In contrast, models 3 and 4 allowed the residual variance to differ by combinations of population and sex. Specifically, in the latter two models, the standard deviation of the residuals (sigma) was modeled as a linear function of the population-by-sex groups. This meant that rather than assuming a single, uniform residual variance, these models could estimate a separate residual variance component, i.e., for females from population A versus males from population B. By explicitly modeling this group-level heterogeneity in residual variability, the third and fourth models provided a more flexible structure that could capture differences in within-individual consistency across the different categories.

All behavioral variables were standardized prior to analysis to facilitate comparisons. All variables, excluding distance moved in the familiar environment, were transformed to fit a Gaussian distribution. All the models were run via Gaussian likelihood, with weakly informative priors specified to constrain the model fitting process. For model selection and comparison, we used the widely applicable information criterion (WAIC) and leave-one-out cross-validation (LOO) [42]. WAIC and LOO showed that for the variables distance moved and angular velocity, Model 3 was the best fit, whereas for the central zone duration, Model 1 was the best fit (Table S1). We calculated repeatability, defined as the ratio of total phenotypic variation in a behavioral trait that can be attributed to consistent differences among individuals (i.e., among-individual variation), according to Eq. 1 [43]:

$$R=\frac{{\text{V}}_{A}}{{\text{V}}_{A}+ {\text{V}}_{W}}$$

Among-individual variation (Va) refers to behavioral differences among individuals within a population. High among-individual variation indicates more behavioral diversity, whereas low among-individual variation indicates that individuals in the population are rather behaviorally homogenous. Within-individual variation (Vw) refers to the variability in behavior observed within the same individual over time or context. This is frequently associated with behavioral flexibility, which allows individuals to adapt their behavior in response to changing environmental conditions. Statistical analyses were performed in the R statistical environment, utilizing packages car [44], lme4 [45], DescTools [46], and multcomp [47] for statistical modeling and multiple comparisons; brms [48] for Bayesian regression analyses; dplyr [49] and tidyr [50] for data manipulation and restructuring; ggplot2 [51] and gridExtra [52] for visualization; MASS [53] for additional statistical procedures; and bayesplot [54] for the diagnostics and visualization of Bayesian models.

## Results

When each of the measured behaviors was analyzed separately at the population by sex average level, we found no significant differences in the activity/exploration traits distance moved and angular velocity, nor in the boldness trait central zone between the populations and sexes (Table 2, Fig S3).

**Table 2:** The effect size and 95% credible intervals of the difference in the average level of activity/exploration behavioral traits (distance moved, angular velocity) and boldness (central zone) per population, sex and population by sex

Distance moved	Estimate	Lower 95%	Upper 95%
Population	0.04	− 0.35	0.41
Sex	− 0.16	− 0.5	0.18
Population*Sex	0.35	− 0.16	0.86

Angular velocity			
Population	− 0.1	− 0.52	0.31
Sex	− 0.26	− 0.59	0.08
Population*Sex	0.26	− 0.28	0.8

Central zone			
Population	− 0.1	− 0.58	0.4
Sex	0.26	− 0.18	0.7
Population*Sex	− 0.19	− 0.83	0.46

Although these findings indicate similar levels of behaviors across populations and sexes, the subsequent variance analysis revealed behavioral differences between populations.

**Fig. 1:**
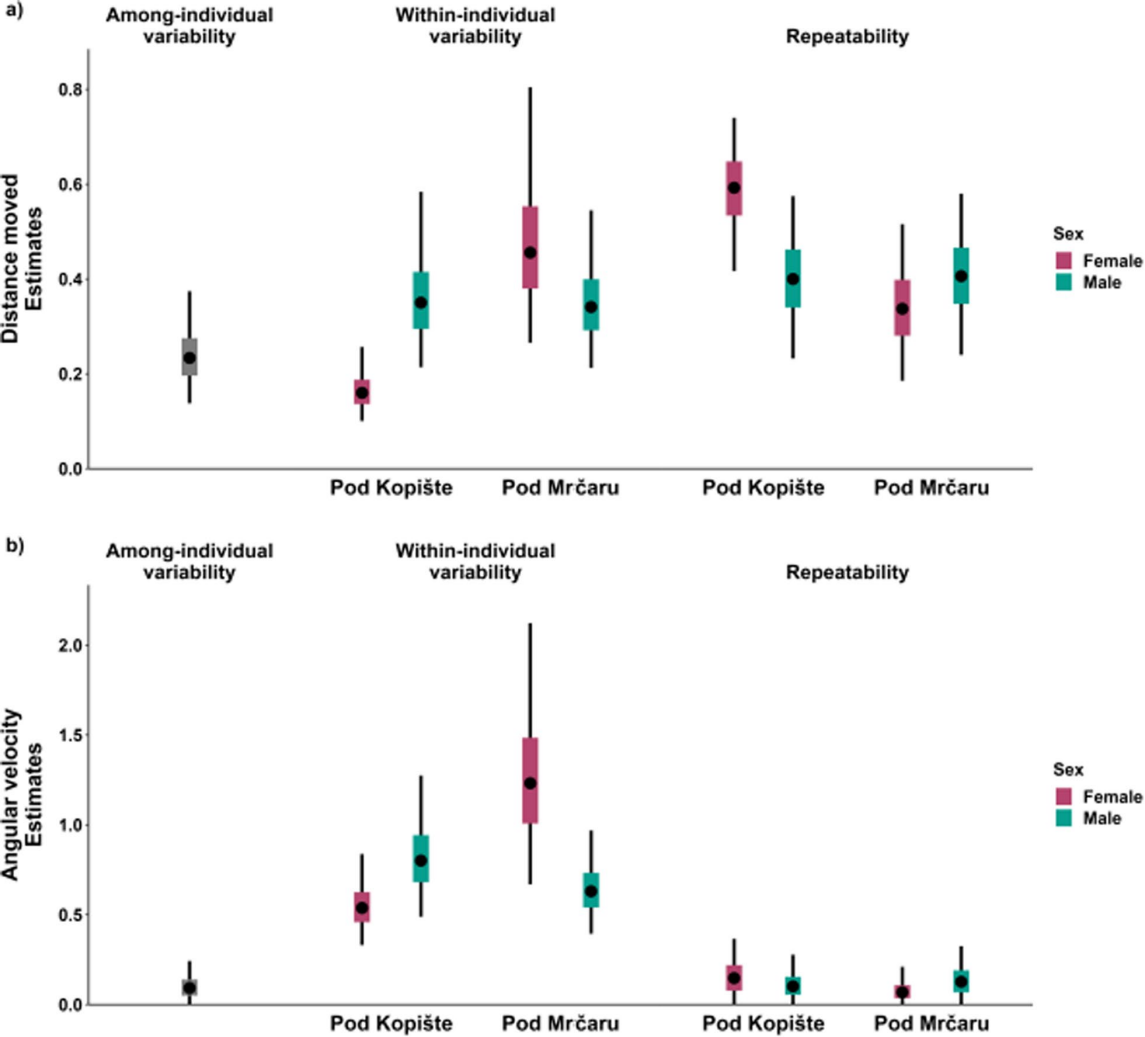
Estimates of among- (Va) and within- (Vw) individual variance and repeatability (R) of activity/exploration behavioral traits **a)** distance moved and **b)** angular velocity in populations by sex groups of *Podarcis siculus* from islands of Pod Kopište and Pod Mrčaru. The plots show the mean (circle), 25–75% credible intervals (color box), and 95% credible intervals (whiskers)

We used variance partitioning to determine whether the transfer of lizards to the new environment led to altered behavioral variance among and within individuals in the newly established population, following a single propagule of 10 individuals. The model with the best fit for distance moved and angular velocity (variables describing activity/exploration) allowed only within-individual variance to vary among population by sex groups (Model 3, Table S1), which means that there were no substantial differences in among-individual variation between them (Fig. 1).

The best fitted model for the variable central zone (boldness) did not allow for within-individual or among-individual variance to differ (Model 1, Table S1), meaning that no substantial differences in either variance component in boldness exists among groups.

Compared with the ancestral population on Pod Kopište, the population on Pod Mrčaru presented different relationships between sexes in terms of within-individual variation in distance moved and angular velocity (Fig. 1). Within-individual variability in activity/exploration behavioral traits strongly increased in females on Pod Mrčaru for both traits, while in males didn’t change substantially (Fig. 1, Table 3).

**Table 3:** The effect size estimates and 95% credible intervals of the difference in within-individual variation (ΔVw, above diagonal) and repeatability (ΔR, below diagonal) of the activity/exploration behavioral traits distance moved and angular velocity between populations by sex groups of *Podarcis siculus* from Pod Kopište and Pod Mrčaru

	Δ Vw				
	Group	PKF	PKM	PMF	PMM
Distance moved					
Δ R	PKF		− **0.20 (**− **0.42,** − **0.03)**	− **0.31 (**− **0.64,** − **0.09)**	− **0.19 (**− **0.39,** − **0.03)**
	PKM	**0.19 (0.03, 0.34)**		− 0.12 (− 0.47, 0.18)	0.01 (− 0.22, 0.26)
	PMF	**0.25 (0.09, 0.40)**	0.06 (− 0.10, 0.22)		0.13 (− 0.15, 0.48)
	PMM	**0.18 (0.03, 0.33)**	− 0.01 (− 0.15, 0.14)	− 0.07 (− 0.22, 0.09)	

	Δ Vw				
	Group	PKF	PKM	PMF	PMM
Angular velocity					
Δ R	PKF		− 0.27 (− 0.73, 0.11)	− **0.72 (**− **1.55,** − **0.13)**	− 0.10 (− 0.44, 0.23)
	PKM	NA		− 0.45 (− 1.32, 0.23)	0.18 (− 0.25, 0.66)
	PMF	NA	NA		**0.63 (0.01, 1.46)**
	PMM	NA	NA	NA	

PKF – female lizards from Pod Kopište; PKM – male lizards from Pod Kopište; PMF – female lizards from Pod Mrčaru; PMM – male lizards from Pod Mrčaru. All substantially different comparisons are highlighted in bold

Substantial sexual dimorphism was observed for within-individual variability in distance moved on Pod Kopište (where males exhibited higher values) and for angular velocity on Pod Mrčaru (where females displayed higher values than males). In all the groups, the trait distance moved was repeatable, whereas angular velocity was not, due to a substantially lower ratio of among-individual to within-individual variation (Fig. 1). As among-individual variation was constant, repeatability of distance moved was reciprocal to within-individual variation, with the highest values observed for Pod Kopište females (Fig. 1, Table 3). The model with the best fit for boldness (central zone, Model 1) demonstrated no differences in within- and among-individual variability or in repeatability across population by sex groups (Fig. S4), indicating that variability in boldness is fixed between environments. Among-individual variation in boldness was higher than within-individual, resulting in substantial repeatability.

## Discussion

Our goal was to explore the behavior and behavioral variability of a newly established lizard population on Pod Mrčaru, for which propagule pressure and the absence of selective filters during the transport and introduction phase were documented. Given the established differences in environmental factors (diet, vegetation, shelter, and predators) and the confirmed phenotypic divergence between the Pod Kopište and Pod Mrčaru populations [33–35, 55, 56], we expected them to also differ in activity/exploration behavioral traits and boldness. However, we observed no substantial differences in those behaviors between populations, thus rejecting our first hypothesis.

Small propagule size and a low number of introduction events can decrease variability in populations [26]. Additionally, selective filters during the introduction process can result in a further reduction in behavioral variation among individuals compared with the source population [4]. Our results on variance partitioning revealed no difference among population by sex groups in among-individual variation for distance moved or central zone (boldness). Furthermore, among-individual variation in angular velocity was negligible (not significantly different from zero) across all groups. Thus, we reject our prediction of a decrease in the variation in behavioral traits among the Pod Mrčaru individuals. The absence of a decrease in the average level and among-individual variation in distance moved and boldness between the ancestral (Pod Kopište) and new (Pod Mrčaru) populations could be attributed to the lack of a strong founder effect. It appears that the founding individuals harbored behavioral variation representative of the ancestral population. This finding is in accordance with our prior research, which showed that the introduction of only 10 individuals did not have a significant bottleneck effect on morphological traits or the genetic background of the Pod Mrčaru population [35]. Furthermore, no additional propagule events occurred, which could have positively influenced the behavioral variability among individuals [26]. Notably, Pod Mrčaru island is characterized by a less diverse prey community [35]. Such environmental conditions provide less opportunity for specialization [57, 58] and thus are unlikely to support the increase in among-individual variability in activity/exploration behavior.

In contrast, within-individual variation in activity/exploration behavioral traits (distance moved and angular velocity) differed between the Pod Kopište and Pod Mrčaru populations, with an apparent effect of sexual dimorphism, while no such differences were observed for boldness. These results agree with our predictions of increased within-individual variation in activity/exploration behavior and the lack of change in boldness. Such context-dependent within-individual variation in behavior allows fast adaptation to novel environmental conditions [18]. In particular, the increase in within-individual variability in activity/exploration behavior can enable animals to more efficiently acquire resources [13]. For example, reef manta rays exhibit high within-individual variation in foraging behavior as an adaptation to the ephemeral nature of zooplankton upwellings, increasing their feeding efficiency [59]. Behavioral flexibility is also known to aid in the establishment of invasive populations of birds by facilitating the adoption of novel food resources or changes in antipredator strategies in new environments [9].

Our results revealed a strong impact of sexual dimorphism on lizards' activity/exploration behavioral traits, with sex-dependent variation not only at the population level but also in the direction and magnitude of change in variability in a new environment. Adaptive plasticity is often sex specific, especially when there are differences in environmentally conditioned phenotypic optima [60]. Males often tend to be more variable in activity and exploratory behaviors, both among and within individuals (e.g. mice [61, 62]). Furthermore, while we detected no difference in behavioral variance among males or females, among-individual variability in locomotion, exploration of novel environments, and predator response were previously found to be generally higher in male than female lizards of genus *Podarcis* [63]. When *P. siculus* was introduced on Pod Mrčaru, the island was inhabited by *P. melisellensis* [30] and given their similar dietary niches, the two species were in high competition for resources*.* In the early phase of *P. siculus* population establishment, reproductive output must have been an important factor in competition with native *P. melisellensis*. Females are the determining factor in lizards’ reproductive success and population growth [64], and female dietary input strongly affects egg production [65]. This could have underlie different pressures on activity/exploration behavior between sexes, as slight advantages in energetically demanding female reproductive physiology would exert strong and immediate effects on *P. siculus* population fitness. Furthermore, habitat structure may also play a role in shaping these behaviors. The lower intra-individual variability in females activity/exploration behavioral traits on Pod Kopište might be linked to the fact that on the large surface of that island vegetation is grazed upon by goats and thus low, while Pod Mrčaru is characterized by patches of rocks and taller vegetation, making microhabitats of that island more heterogenous (photos of the islands are available in Supplementary materials of reference 35). Environmental conditions are also known to specifically affect female lizard behavior. For example, meta-study on lizards found that females increase their boldness and thus foraging activity at higher latitudes due to constrained time window to gather enough resources for reproduction [66].

Notably, the relevance of *P. siculus* exploratory behavior for its feeding efficiency and competitive success has been previously shown. For example, competitive interactions between *P. siculus and Podarcis virescences* do not include aggressive interactions. Nonetheless, the more efficient feeding of *P. siculus*, which was attributed to its higher exploratory behavior, contributed to its outcompeting success [67, 68]. Our previous work has also shown that mainland *P. siculus* is more exploratory and voracious than sympatric *P. melisellensis* [36], confirming the difference in behavioral strategies between these two species.

The lack of repeatability in angular velocity is a consequence of among-individual variation not significantly differing from zero. In contrast, distance moved exhibited consistent among-individual variation across groups, making its repeatability dependent primarily on within-individual variation [43]. Specifically, as within-individual variation increases, repeatability decreases.

Intriguingly, our results revealed a lack of differences in boldness between the ancestral and novel populations, both in the terms of population-by-sex average and among- and within-individual variability across groups. Boldness is known to play a significant role in introduction scenarios through selection in the transport phase [4], which was circumvented in our system. A decrease in boldness is expected to aid survival in new habitats that include a substantial increase in predator pressure or predator novelty, whereas greater boldness is expected to be preferable in environments with lower predator pressure, as the animals can better exploit resources and allocate less time to predator vigilance [4, 69, 70]. Although predator pressure on Pod Mrčaru is slightly reduced [34], gulls, the predominant bird species on both islands, are not considered genuine lizard predators. Instead, they sporadically engage in opportunistic attacks that are seldom successful [71, 72]. Hence, the overall expected predator pressure on both islands is relatively low, which explains the lack of selective pressure on boldness. Conversely, Vervust et al. [34] reported lower antipredator behavior (in terms of approach and fleeing distances) in lizards from Pod Mrčaru. However, the measured traits related to boldness are different between these two studies. Moreover, Vervust et al. [34] studied behaviors in situ, meaning that environmental settings between the two populations, including the vegetation, open area and shelter availability, differed, while in our laboratory experiment lizards were acclimatized and exposed to the same standardized treatment. Due to uniqueness of our study system, we were not able to find replicate populations in natural setting, and future studies are warranted to evaluate if these findings are specific to this populations or represent widespread adaptive mechanisms in other novel lizard populations.

## Conclusion

Collectively, our findings suggest that while propagule pressure plays a significant role in determining establishment success [25], behavioral variation can be conserved even under conditions of very small propagule size. This study specifically emphasizes the significant role of within-individual variation in adaptive responses to novel environments and underlines sexual differences in establishment strategies. The lack of differences in among-individual variation in all the studied behaviors suggests that the among- and within-individual variation could be selected independently from one another. Additionally, the persistence of these differences in behavioral flexibility after six months of laboratory acclimatization indicates that they may reflect underlying genetic or developmental differences rather than being directly conditioned by the environment. In conclusion, these results highlight the importance of studying not only population and individual average behaviors but also behavioral flexibility in adapting to new environments. Our study also revealed that specific environmental conditions differentially influence the variation in particular behavioral traits and emphasized the importance of variance in female activity/exploration behavior for reproductive success at the early stages of population establishment.

## Supplementary Information

Additional file 1.

## Data Availability

The datasets and R code used and/or analyzed during the current study are available on 10.6084/m9.figshare.29589851.
