# Supplementary material for: Sex-specific behavioral flexibility in rapid adaptation to a new environment
Source: Front Zool. 2025 Nov 4;22:32. doi: 10.1186/s12983-025-00586-y (PMC12584423; doi:10.1186/s12983-025-00586-y)
Supplement: Supplementary file 1 — Additional file 1. [file 12983_2025_586_MOESM1_ESM.docx]

**
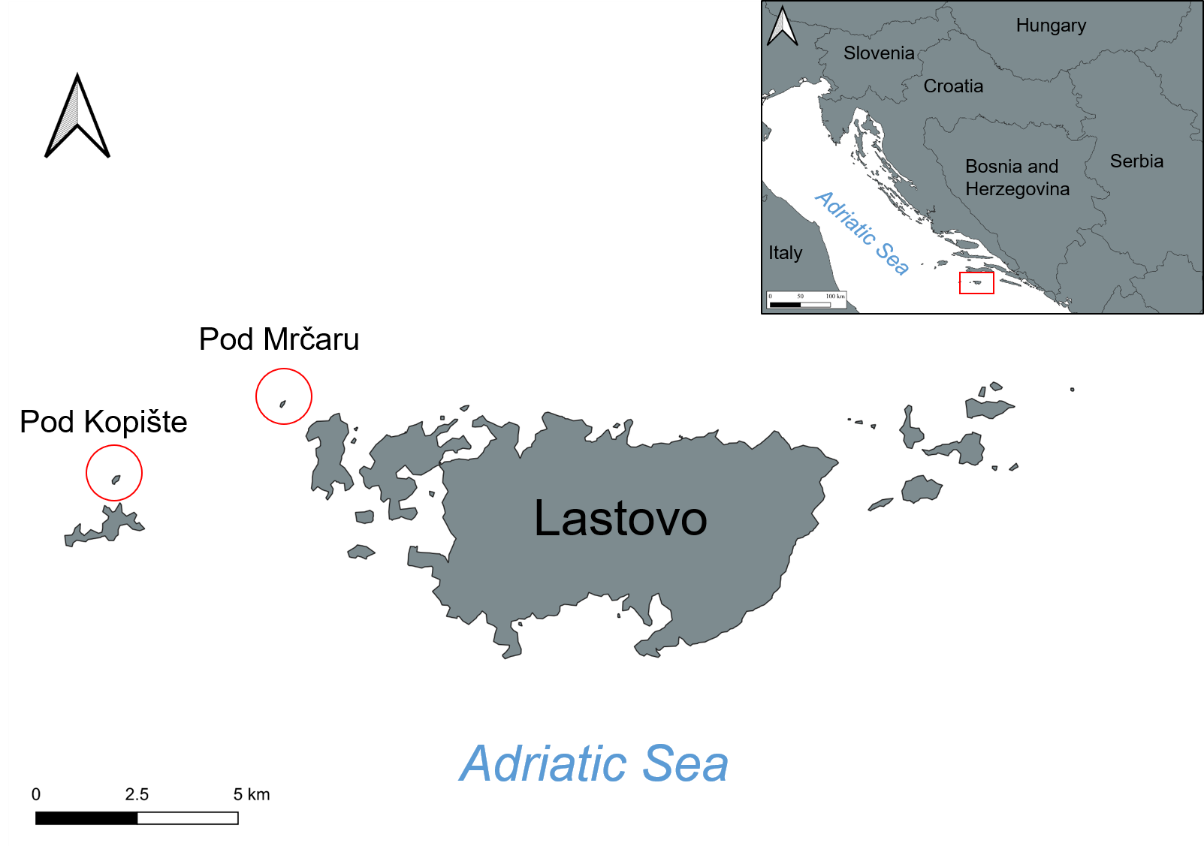
**

**Figure S1.** Map of the Lastovo Archipelago showing the islands Pod Kopište and Pod Mrčaru.


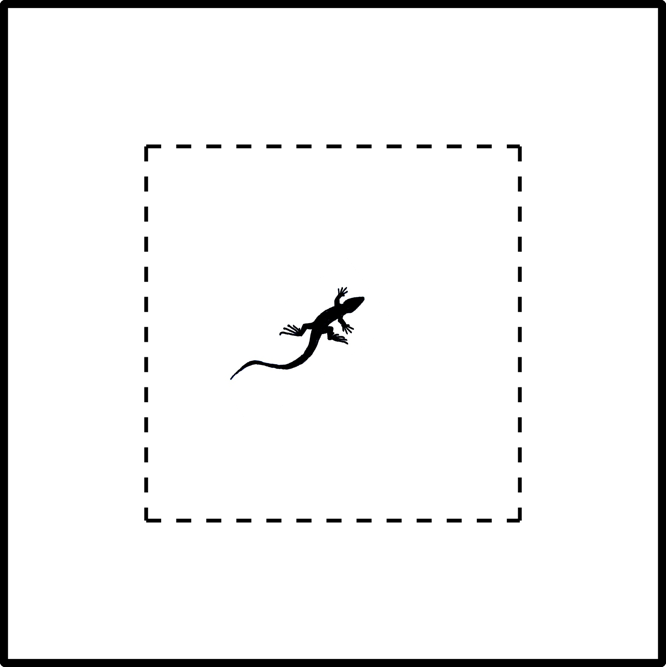


**Figure S2.** Diagram of open field (50 x 50 x 50 cm). Dashed line represents the border between central and peripheral zone (10 cm from the wall).

**Table S1**. Widely Applicable Information Criterion (WAIC) and Leave-One-Out Cross-Validation Information Criterion (LOOIC) results of model selection for all behavioural variables. Bold values indicate the best model.

| WAIC comparison | **Distance moved** | | **Angular velocity** | | **Central zone** | |
| --- | --- | --- | --- | --- | --- | --- |
|  | Expected log predictive density ± SE | WAIC ± SE | Expected log predictive density ± SE | WAIC ± SE | Expected log predictive density ± SE | WAIC ± SE |
| Model 1 | -251.24 ± 11.83 | 502.48 ± 23.66 | -379.48 ± 17.27 | 758.95 ± 34.53 | **-296.01 ± 11.30** | **592.02 ± 22.61** |
| Model 2 | -254.56 ± 11.87 | 509.13 ± 23.74 | -380.85 ± 17.77 | 761.70 ± 35.53 | -297.27 ± 11.71 | 594.55 ± 23.42 |
| Model 3 | **-246.73 ± 11.56** | **493.46 ± 23.13** | **-374.42 ± 14.17** | **748.84 ± 28.35** | -296.04 ± 11.06 | 592.09 ± 22.11 |
| Model 4 | -249.15 ± 11.66 | 498.31 ± 23.32 | -375.02 ± 14.07 | 750.04 ± 28.15 | -297.56 ± 11.50 | 595.13 ± 23.01 |
| LOOIC comparison | Expected log predictive density ± SE | LOOIC ± SE | Expected log predictive density ± SE | LOOIC ± SE | Expected log predictive density ± SE | LOOIC ± SE |
| Model 1 | -254.46 ± 12.14 | 508.91 ± 24.28 | -380.42 ± 17.75 | 760.83 ± 35.51 | **-299.90 ± 11.64** | **599.81 ± 23.27** |
| Model 2 | -257.90 ± 12.18 | 515.80 ± 24.36 | -382.93 ± 18.91 | 765.87 ± 37.83 | -300.89 ± 11.94 | 601.79 ± 23.88 |
| Model 3 | **-250.66 ± 11.93** | **501.33 ± 23.85** | **-375.70 ± 14.50** | **751.40 ± 29.00** | -300.88 ± 11.53 | 601.76 ± 23.06 |
| Model 4 | -253.37 ± 12.03 | 506.75 ± 24.05 | -377.29 ± 14.72 | 754.58 ± 29.44 | -301.97 ± 11.86 | 603.95 ± 23.73 |


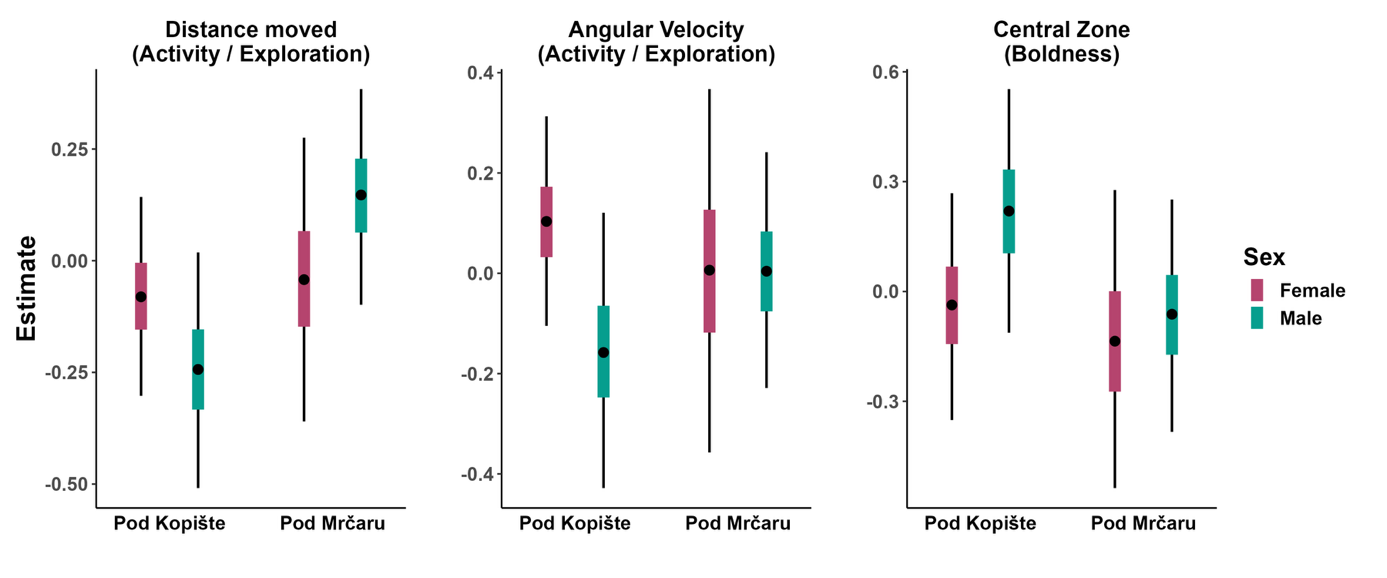


**Figure S3**. Estimates of activity/exploration behavioural traits (distance moved, angular velocity) and boldness (central zone) in populations by sex groups of *Podarcis siculus* from islands of Pod Kopište and Pod Mrčaru. The plots show mean (circle), 25–75 % credible intervals (color box), and 95 % credible intervals (whiskers).


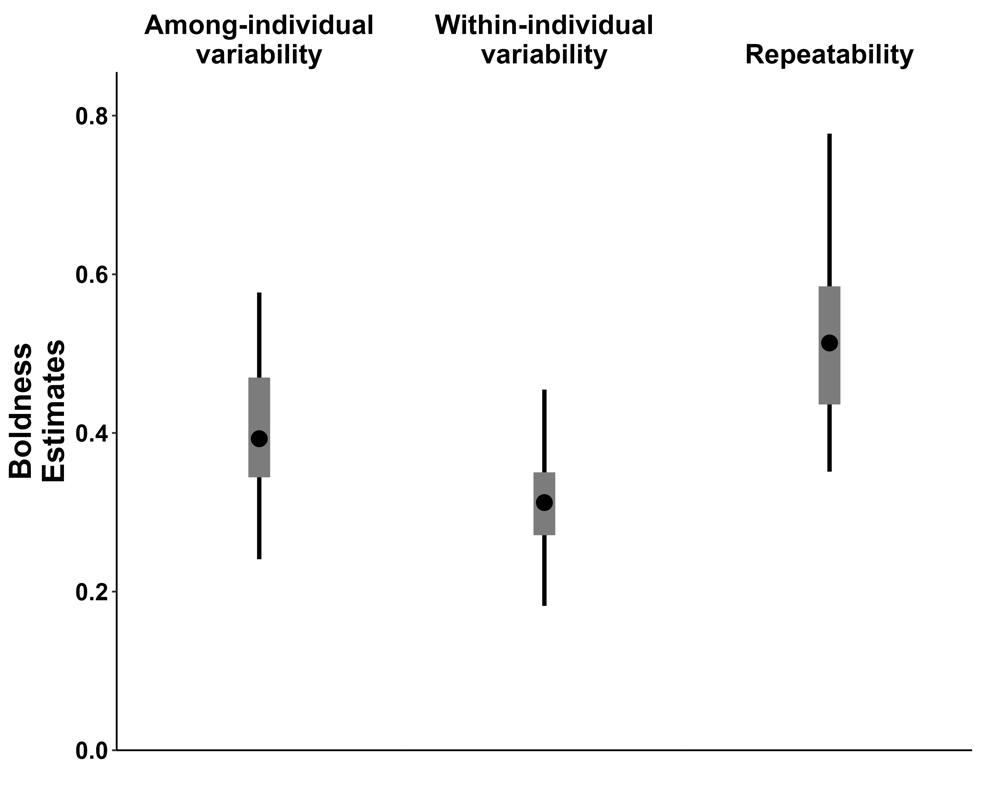


**Figure S4**. Estimates of among- (Va) and within- (Vw) individual variance, and repeatability (R) of boldness (central zone) in *Podarcis siculus* from islands of Pod Kopište and Pod Mrčaru. The plot shows median (circle), 25–75 % confidence intervals (grey box), and 95 % confidence intervals (whiskers).
